# Profiling the proteomic inflammatory state of human astrocytes using DIA mass spectrometry

**DOI:** 10.1186/s12974-018-1371-6

**Published:** 2018-11-30

**Authors:** Vito Dozio, Jean-Charles Sanchez

**Affiliations:** 1 Department of Internal Medicine Specialties, Faculty of Medicine, University of Geneva, 1 Rue Michel Servet, 1211 Geneva 4, Switzerland; 2 Swiss Centre for Applied Human Toxicology, Geneva, Switzerland

**Keywords:** Astrocytes, DIA MS, Proteomics, Inflammation, Blood-brain barrier

## Abstract

**Background:**

Astrocytes are the most abundant cells in the central nervous system and are responsible for a wide range of functions critical to normal neuronal development, synapse formation, blood-brain barrier regulation, and brain homeostasis. They are also actively involved in initiating and perpetuating neuroinflammatory responses. However, information about their proteomic phenotypes under inflammation is currently limited.

**Method:**

Data-independent acquisition mass spectrometry was applied to extensively characterize the profile of more than 4000 proteins in immortalized human fetal astrocytes under distinct inflammatory conditions induced by TNF, IL-1β, and LPS, while multiplex immunoassay-based screening was used to quantify a wide range of cytokines released under these inflammatory conditions. Then, immunocytochemistry and western blotting were used to verify the activation of canonical and non-canonical NF-κB upon exposure to the different stimuli. Finally, an in vitro model of the blood-brain barrier consisting of a co-culture of primary human brain microvascular endothelial cells and primary human astrocytes was used to verify the inflammatory response of astrocytes upon LPS exposure in a more complex in vitro system.

**Results:**

We reported on a set of 186 proteins whose levels were significantly modulated by TNF, IL-1β, and LPS. These three stimuli induced proteome perturbations, which led to an increased abundance of key inflammatory proteins involved in antigen presentation and non-canonical NF-κB pathways. TNF and IL-1β, but not LPS, also activated the canonical NF-κB pathway, which in turn led to an extensive inflammatory response and dysregulation of cytoskeletal and adhesion proteins. In addition, TNF and LPS, but not IL-1β, increased the abundance of several interferon-stimulated gene products. Finally, TNF and IL-1β similarly upregulated the secretion of several cytokines and chemokines, whereas LPS only induced a moderate increase in IL-8, IFN-γ, and IL-1β secretion. Upregulation of proteins associated with type I IFN and non-canonical NF-κB signaling was also observed in primary astrocytes co-cultured with primary brain microvascular endothelial cells exposed to LPS.

**Conclusions:**

The present study provides comprehensive information about the proteomic phenotypes of human astrocytes upon exposure to inflammatory stimuli both in monoculture and in co-culture with human brain microvascular endothelial cells.

**Electronic supplementary material:**

The online version of this article (10.1186/s12974-018-1371-6) contains supplementary material, which is available to authorized users.

## Background

A successful inflammatory response is essential for eliminating invading pathogens and for promoting angiogenesis and wound healing inside the brain. Prolonged inflammation of the central nervous system (CNS), however, can lead to detrimental consequences and is linked to several classic neurodegenerative diseases, such as multiple sclerosis [1], Alzheimer’s disease (AD) [2], and Parkinson’s disease (PD) [3].

Astrocytes are the most abundant glial cell type and constitute about 40% of all the cells in the human brain. Their roles span maintaining brain homeostasis [4], supporting synapse formation and plasticity [5], regulating the extracellular balance of ions and neurotransmitters, and maintaining the blood-brain barrier (BBB) [6]. Astrocytes also play such important roles as controlling immune cell trafficking and activation through the secretion of cytokines and chemokines [7] as well as antigen-presenting cells [8]. During insults to the CNS, astrocytes undergo a substantial transformation, producing glial scars in a process commonly known as “reactive astrogliosis.” Such astrocytic activation results in the expression of structural proteins (e.g. glial fibrillary acidic protein (GFAP) and vimentin) and adhesion molecules. The functional role of reactive astrogliosis remains largely unknown as both detrimental (inhibition of axon regeneration after CNS injury) and beneficial (improving recovery after CNS trauma and restriction of inflammation during infections) consequences have been associated with it [9]. The hypothesis of the dual nature of astrocytes is reinforced by studies indicating two subtypes of reactive astrocytes, termed *A1* and *A2*, able to induce neurotoxic and neuroprotective effects, respectively [10].

Cytokines contribute to nearly all aspects of neuroinflammation. They are usually key mediators of astrogliosis, crucial for the evolution of neuropathological changes [11], and typically more elevated in neurodegenerative disorder states [12]. IL-1β is an acute-phase cytokine, generally produced in response to cell damage, and inside the brain, it is mostly released by microglia. High concentrations of IL-1β are detected in the microglial cells surrounding Aβ plaques in the brains of patients with AD [13] and in the post-mortem brains, serum, and cerebrospinal fluid of PD patients [14]. TNF is one of the most important and prototypical pro-inflammatory cytokines, and it is commonly found at higher concentrations in patients (and animal models) with several CNS pathologies [15]. Both IL-1β and TNF are involved in recruiting various peripheral immune cells into the brain, and they are implicated in the development and maintenance of neuropathic pain after peripheral nerve injury or infection [16].

Astrocytes are commonly known to be activated by microglia, but they are also able to respond directly to infections initiated by parasites [17], viruses [18], and bacteria [19]. However, the ability of human astrocytes to respond directly to lipopolysaccharide (LPS)—a structural component of the outer envelope of all gram-negative bacteria—is still in debate [20, 21].

Most cellular inflammatory responses are mediated by the NF-κB transcription factor. Five members of the NF-κB family exist: RelA (p65), RelB, c-Rel, NFKB1 p50/p105, and NFKB2 p52/p100. NF-κB proteins bind as dimers to κB sites in the promoters and enhancers of several genes, inducing or repressing their transcription [22]. Activation of NF-κB can be induced via canonical and non-canonical (or alternative) pathways. The canonical pathway commonly involves the nuclear translocation of p50/RelA heterodimers and regulates a potent inflammatory response, whereas the non-canonical pathway involves the processing of NFKB2 p100 and the nuclear translocation of the p52/RelB heterodimer and is thought to regulate immune cell differentiation, attenuate apoptosis, and be associated with less acute and chronic inflammation [23]. NF-κB activation is needed for correct innate and adaptive immune responses, but it can lead to acute and chronic inflammatory diseases and tumorigenesis when deregulated [24]. In astrocytes, activation of NF-κB and NF-κB-dependent transcription factors is associated with astrogliosis and neurodegenerative processes [25].

Most of the current knowledge about glial biology is based on mouse models and is at the transcript level—there is very little information available at the protein level. Proteins essentially regulate every cellular process, and their abundance adapts dynamically to external and internal perturbations and thus defines the functional states and phenotypes of cells. The high-throughput quantitative analysis of proteins is therefore a powerful tool to better understand the molecular mechanisms of diseases.

In the present study, we applied data-independent acquisition mass spectrometry (DIA MS) [26] to systematically profile the abundance of more than 4000 proteins in immortalized human fetal astrocytes exposed to TNF, IL-1β, and LPS. In addition, the cytokines and chemokines released by those stimulations were screened in supernatants. We discovered several clusters of proteins whose abundance was differently modulated upon exposure to the inflammatory stimuli. We found evidence that TNF and LPS, but not IL-1β, were able to increase the abundance of several interferon-inducible proteins and that LPS predominantly activated NF-κB through the non-canonical pathway. Finally, we verified the abundance of several inflammatory-related proteins in primary human astrocytes co-cultured with primary human brain microvascular endothelial cells in an in vitro model of the blood-brain barrier exposed to LPS and found a similar pattern of astrocyte activation.

## Methods

### Cell culture and treatments

Immortalized human fetal astrocytes (Applied Biological Materials Inc.) were seeded onto rat tail collagen type I-coated plates (15 μg/ml, Merck Millipore) at 5000 cells/cm^2^ and maintained in Prigrow IV medium (Applied Biological Materials Inc.) supplemented with 10% heat-inactivated FBS (Gibco), 100 U/ml penicillin, and 100 μg/ml streptomycin (Gibco) at 37 °C in 5% CO_2_. The medium was replaced every other day. At 80% confluence, cells were treated, in triplicates, with TNF (Enzo Life Sciences) (100 ng/ml), IL-1β (Enzo Life Sciences) (100 ng/ml), and ultra-pure LPS-EK (InvivoGen) (10 μg/ml) for 24 h or as indicated. Cells were detached using StemPro Accutase (Gibco), washed three times with ice-cold phosphate-buffered saline (PBS, Gibco), and dry-stored at − 80 °C.

### In vitro blood-brain barrier model and LPS exposure

Primary human brain microvascular endothelial cells (HBMECs) (ScienCell) were grown in complete endothelial cell growth medium (EGM-2MV) (Lonza) while primary human astrocytes (ScienCell) were maintained in astrocyte medium (ScienCell). The in vitro BBB model was established as described here [27]. Transwell PET membrane inserts (12 mm diameter, 0.4 μm pore size) (Corning) were coated with rat tail collagen type I (15 μg/ml) (Merck Millipore) and fibronectin (30 μg/ml) (Sigma-Aldrich) on the apical side and with poly-l-lysine (20 μg/ml) (ScienCell) on the basolateral side. Inserts were turned upside down, and primary human astrocytes were seeded at a density of 25,000 cells per insert and let adhere overnight. Subsequently, inserts were reversed and HBMECs were seeded, on the apical side, at a density of 200,000 cells per insert. The culture was maintained at 37 °C in 5% CO_2_ for 4 days.

Ultra-pure LPS-EK (100 ng/ml final concentration) (InvivoGen) was added into the apical chamber. Upon 24-h exposure, astrocytes were detached from the basolateral side of the Transwell insert using Accutase (Gibco), washed three times with ice-cold PBS (Gibco), and dry-stored at − 80 °C. All the experiments were performed in triplicates.

### Sample preparation for mass spectrometry-based proteomics

Cell pellets were resuspended in 0.1% RapiGest (Waters) and 100 mM TEAB (Sigma-Aldrich), sonicated (5 cycles of 20 s with breaks on ice), and incubated for 10 min at 80 °C. Samples were then spun down (14,000*g*, 10 min, 4 °C), the supernatant was recovered, and the protein content was measured using the Bradford assay (Bio-Rad).

For each sample, 20 μg of proteins was reduced using TCEP (final concentration 5 mM, 30 min, 37 °C) (Sigma-Aldrich), alkylated using iodoacetamide (final concentration 15 mM, 60 min, RT, in dark conditions) (Sigma-Aldrich), and digested overnight using trypsin (*w*/*w* ratio 1:50) (Promega). The RapiGest surfactant was cleaved by incubating samples with 0.5% trifluoroacetic acid (Sigma-Aldrich) (45 min, 37 °C). Samples were desalted on C18 reverse phase columns (Harvard Apparatus), peptides were dried under vacuum and subsequently resuspended in 5% ACN 0.1% FA, and iRT peptides (Biognosys) were added (1:20).

### Peptide fractionation by Off-Gel electrophoresis

Peptides were separated into 12 fractions according to their p*I* using a 3100 OFFGEL Fractionator (Agilent Technologies). Peptides digested from 120 μg of proteins were solubilized in Off-Gel electrophoresis (OGE) buffer and subsequently focused using an immobilized pH gradient (IPG) strip of 13 cm (pH 3–10) (GE Healthcare). After recovering the fractions, samples were desalted on C18 reverse phase columns (Harvard Apparatus), dried under vacuum, and re-solubilized in 5% ACN and 0.1% FA, and iRT peptides (Biognosys) were added (1:20).

### MS acquisitions

The equivalent of 2 μg of peptides was analyzed using liquid chromatography-electrospray ionization-MS/MS (LC-ESI-MS/MS) on an Orbitrap Fusion Lumos Tribrid mass spectrometer (Thermo Fisher Scientific) equipped with a Thermo EASY-nLC. Peptides were trapped on a 2 cm × 75 μm i.d. PepMap C18 precolumn packed with 3 μm particles and 100 Å pore size. Separation was performed using a 50 cm × 75 μm i.d. PepMap C18 column packed with 2 μm and 100 Å particles and heated at 50 °C. Peptides were separated using a 125-min segmented gradient of 0.1% FA (solvent A) and 80% ACN 0.1% FA (solvent B) (Additional file 1), at a flow rate of 250 nl/min.

For the spectral library generation, the instrument was operated in data-dependent acquisition (DDA) mode. Full-scan MS was acquired in the Orbitrap with a resolution of 120,000 (FWHM) at m/z 200, scan range of 400–1250 m/z, AGC target of 4 × 10^5^, and a maximum injection time of 50 ms. The top 20 most intense ions were quadrupole-isolated (isolation window 1.4 m/z), fragmented by higher energy collisional dissociation (collision energy 30%), and detected in the Orbitrap with a resolution of 15,000 (FWHM) at m/z 200, AGC target 5 × 10^5^, and a maximum injection time 50 ms. Full-scan MS was acquired in profile mode, whereas MS/MS spectra were acquired in centroid mode.

Immortalized astrocytes were analyzed by data-independent acquisition (DIA) as follows: a full-scan MS was acquired in the Orbitrap with a resolution of 60,000 (FWHM) at m/z 200, scan range of 400–1250 m/z, a maximum injection time 100 ms, and AGC target 10^6^. Then, 40 DIA variable windows (Additional file 2, Method A) were acquired in the Orbitrap with a resolution of 15,000 (FWHM) at m/z 200, AGC target 5 × 10^5^, and maximum injection time 50 ms. Primary astrocytes were analyzed as follows: a full-scan MS was acquired in the Orbitrap with a resolution of 60,000 (FWHM) at m/z 200, scan range of 400–1250 m/z, a maximum injection time 100 ms, and AGC target 10^6^. Then, 30 DIA variable windows (Additional file 2, Method B) were acquired in the Orbitrap with a resolution of 30,000 (FWHM) at m/z 200, AGC target 2 × 10^6^, and maximum injection time 80 ms. The sizes of variable windows were optimized using the swathTUNER software [28].

### Spectral library generation, DIA MS data, and statistical analysis

Spectral libraries were generated using Spectronaut 11 software (Biognosys) [29] by combining the output from DDA MS runs analyzed using Proteome Discoverer 2.0 (Thermo Scientific) and the Mascot search engine. A spectral library for the immortalized astrocytes (42 DDA runs) and a spectral library for the primary astrocytes (12 DDA runs) were generated. The search parameters used included 10 ppm precursor mass tolerance, 0.02 Da fragment mass tolerance, and trypsin miscleavage setting of two. Static modification settings included carbamidomethylation (+ 57.021 Da) on cysteine, whereas dynamic modifications were set to include oxidation (+ 15.995 Da) on methionine. Peptide spectrum matches (PSMs) were verified, based on *q* values set to a 1% false discovery rate (FDR), using the Percolator module. Spectronaut software was subsequently used to match raw DIA MS data against the spectral library, using the default settings with slight modifications. Proteotypicity filter set to only protein group-specific. Data filtering was set to *q* value complete. Cross run normalization was performed by using the *q* value complete setting in row selection. For the analysis of primary astrocytes, protein abundances were exported from Spectronaut, and selected proteins were tested for significance using Student’s two-tailed *t* test. For the analysis of immortalized astrocytes, peptide intensities were exported and analyzed using mapDIA [30]. No further normalization was applied. The following parameters were used: min peptides = 1, max peptides = 5, min correl = − 1, SDF = inf. min obs = 1, Min_DE = 0.01, max_DE = 0.99, and experimental_design = independent design. Proteins were considered to have significantly changed in abundance with an FDR < 0.05 and an absolute fold change (|FC|) > 1.2.

### Cluster, pathway, and correlation analysis

PCA and correlation analysis, as well as hierarchical clustering, were performed using R statistics software [31]. Metacore™ (Thomson Reuters Inc.) was used to map significantly changing proteins onto biological pathways. The top 10 biological pathways were selected.

### Immunocytochemistry

Cells were fixed with 4% paraformaldehyde (PFA) (Santa Cruz Biotechnology) in PBS for 15 min at room temperature, permeabilized with 0.3% Triton X-100 (AppliChem) in PBS for 15 min, and then incubated with 2% bovine serum albumin (BSA) (Sigma-Aldrich) in PBS for 1 h. Fixed cells were then incubated overnight at 4 °C with anti-human NF-κB RelA (Santa Cruz Biotechnology), incubated for 1 h with Alexa 488-conjugated anti-rabbit IgG (Cell Signaling Technology), and finally mounted in Vectashield (Vector Laboratories) with DAPI to counterstain the nuclei.

Confocal images were acquired using LSM 800 laser scanning confocal microscope (Zeiss) using an apochromat × 40/1.4 oil objective (Zeiss) at the Bioimaging Core Facility, Faculty of Medicine, University of Geneva. Image analysis was performed with ImageJ software.

### Western blotting

The equivalent of 10 μg of proteins was resolved by 12% polyacrylamide gel electrophoresis and subsequently transferred onto a PVDF membrane. Immunoblot assays were performed using an anti-human antibody against NFKB1 p105/p50 (Cell Signaling Technology), NFKB2 p100/p52 (Biolegend), and GAPDH (Merck Millipore). Band intensities were retrieved using MYImageAnalysis software (Thermo Scientific) and analyzed using Prism software (GraphPad).

### Multiplex assay for cytokine detection in cell culture supernatants

The concentrations of IFN-α2a, IFN-β, IFN-γ, IL-1β, IL-6, IL-8, IL-10, IL-12p70, and TNF in the cell culture supernatants were evaluated using a multiplex immunoassay on a customized U-plex assay platform (Meso Scale Discovery) according to the manufacturer’s instructions. Cytokine release was analyzed using Prism software (GraphPad).

### MTS and LDH assay

Astrocytes were seeded in a 96-well plate (5000 cells per well) and treated for 24 h with TNF, IL-1β, or LPS. Cell proliferation was determined using the MTS [3-(4,5-dimethylthiazol-2-yl)-5-(3-carboxymethoxyphenyl)-2-(4-sulfophenyl)-2H-tetrazolium] assay (CellTiter 96® AQ_ueous_ One Solution Cell Proliferation Assay, Promega), whereas cytotoxicity was assessed by measuring lactate dehydrogenase (LDH) release using a Pierce™ LDH cytotoxicity kit (Thermo Scientific). Both the MTS and LDH assays were performed according to the manufacturer’s recommendations.

## Results

### Data-independent acquisition mass spectrometry for the identification of the proteomic profiles of inflammation in immortalized human astrocytes

**Fig. 1:**
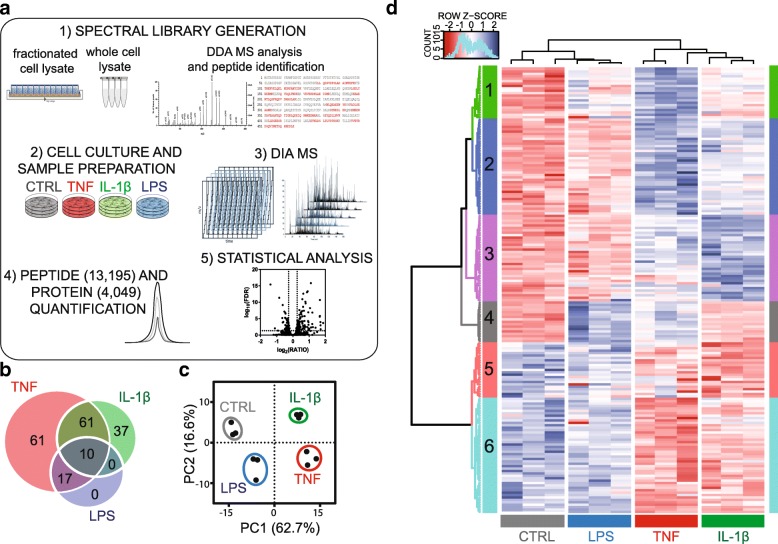
DIA MS workflow and analysis. (**a**) A spectral library was generated using different types of samples. Immortalized human fetal astrocytes were incubated with TNF (100 ng/ml), IL-1β (100 ng/ml), and LPS (10 μg/ml) for 24 h, and protein samples were analyzed using DIA LC-MS. Quantitative data were extracted by matching DIA MS data with the spectral library. Significantly changing proteins (FDR < 0.05, |FC| > 1.2) were selected after statistical analysis. (**b**) Venn diagram displaying significantly changing proteins for each group. (**c**) Principal component analysis (PCA) and hierarchical clustering (**d**) on the abundances of proteins significantly changing in at least one condition

We used DIA MS—a method that allows extensive peptide quantification, with high reproducibility and precision [26]—to study the astrocyte proteome modulation under inflammatory conditions. DIA MS requires an assay library containing the spectra of all the peptides to be quantified. We built a spectral library consisting of 94,377 tryptic peptides belonging to 8684 protein groups by using 42 injections from both unfractionated and fractionated cell lysates derived from immortalized human fetal astrocytes (Fig. 1a).

Human astrocytes were separately exposed, in triplicates, to TNF, IL-1β, and LPS using concentrations that were shown not to induce significant effects on cell proliferation and viability, as measured using the MTS and LDH assays (Additional file 3) but known to induce a potent inflammatory response. After incubation, proteins from the resulting 12 samples (including a triplicate control, CTRL) were extracted and digested, and peptides were analyzed using LC-DIA MS (Fig. 1a). A pool of peptides was injected three times over the whole analysis period to assess the technical reproducibility of the LC-MS analysis. DIA MS allowed the quantification of 13,195 peptides, corresponding to 4049 proteins, with high reproducibility in all samples (median Pearson correlation coefficient of 0.99 for technical replicates, Additional file 4) and with no missing values. High correlations for protein abundance was also observed in the biological replicates (median Pearson correlation coefficient of 0.99, Additional file 4).

### TNF, IL-1β, and LPS-induced proteome modulation

To identify the effects induced by TNF, IL-1β, and LPS, 186 significantly changing proteins were selected after statistical analysis (for full list see Additional file 5). Overall, TNF induced the strongest effects, modulating the abundance of 149 proteins, followed by IL-1β (108 proteins) and LPS (27 proteins) (Fig. 1b). The abundances of 10 proteins were found to commonly change upon exposure to all three stimuli while 71 proteins were common to TNF and IL-1β groups. TNF and IL-1β were found to similarly modify the abundance of these common proteins (squared Pearson correlation coefficient of ratios equal to 0.85, Additional file 6). All of the 27 significantly changing proteins upon exposure to LPS were also found significantly more abundant upon TNF exposure. A principal component analysis (PCA) of the abundances of proteins that were significantly changing under at least one condition assessed whether the proteome modulation patterns were similar between inflammatory conditions and consistent among biological replicates (Fig. 1c). All biological replicates were closely grouped, and the first component (PC1) accounted for most of the variability (62.7%) and clearly separated TNF and IL-1β from CTRL and LPS. Instead, the second component (PC2, 16.6%) grouped LPS and TNF, outdistancing CTRL and IL-1β.

**Fig. 2:**
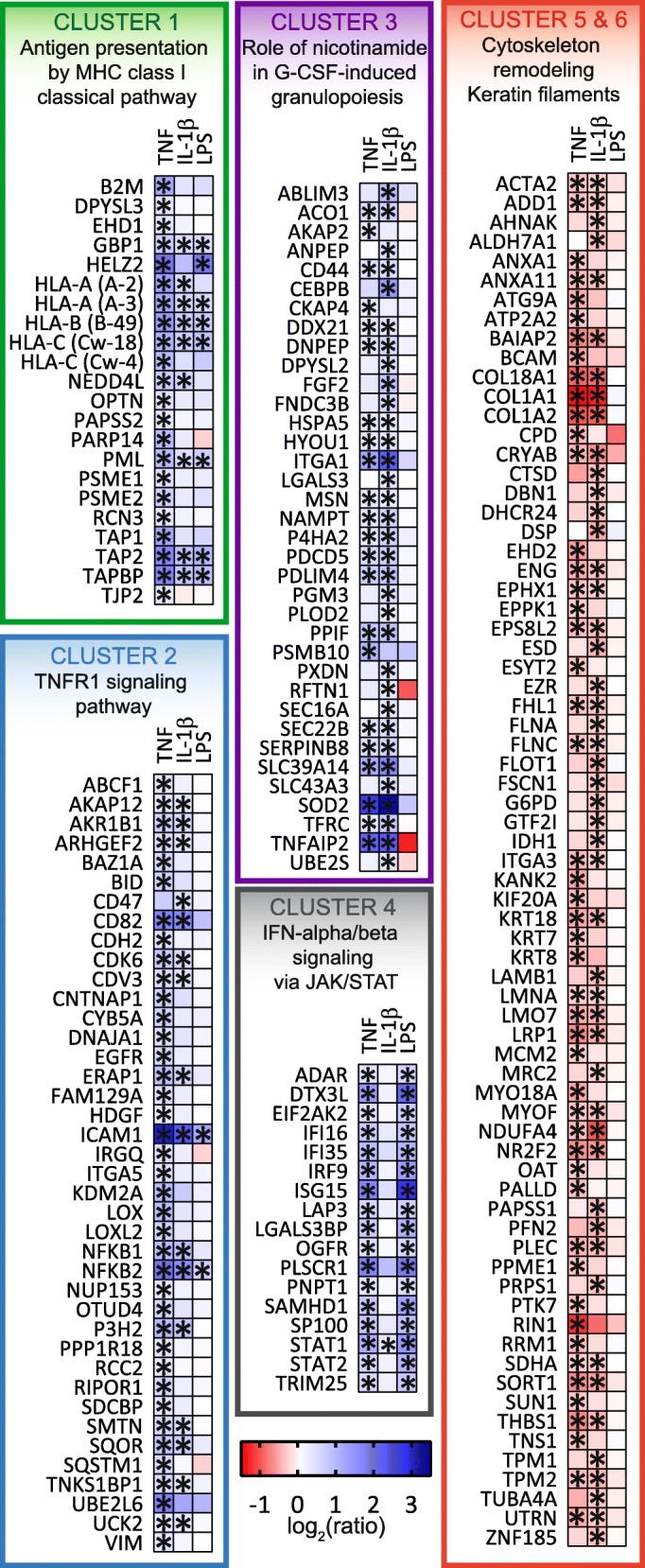
Proteins whose abundance was found to have significantly changed (FDR < 0.05, |FC| > 1.2) under at least one condition (TNF, IL-1β, and LPS) were clustered into six groups using hierarchical clustering. Asterisks represent significance (FDR < 0.05, |FC| > 1.2) for a certain condition. Proteins from each cluster were mapped into biological pathway, and the most significant pathway was represented (for full list, see Additional file 7)

Subsequently, we used hierarchical clustering to group significantly changing proteins into six clusters (Fig. 1d). Proteins belonging to each cluster were mapped onto biological pathways (for top 10 pathways for each cluster, see Additional file 7), and their ratios were displayed using a heat map (Fig. 2).

Cluster 1 grouped several proteins which were significantly more abundant in all three groups. Most of these proteins belonged to the MHC class I antigen presentation pathway, including the three types of HLA class I molecules (HLA-A, HLA-B, HLA-C), antigen peptide transporter (TAP1, TAP2), and tapasin (TAPBP). Other important proteins belonging to this cluster, but not associated with antigen presentation, were the GBP1 (found more abundantly in all groups) and HELZ2 (increased upon TNF and LPS exposure) antiviral proteins.

Proteins belonging to cluster 2 were instead differentially changing upon TNF and, to some extent, IL-1β exposure and were mapped onto the TNRF1 signaling pathway. Several key inflammatory proteins belonged to this cluster, such as ICAM1, SQSTM1, EGFR, NFKB1, and NFKB2. Of particular interest was NFKB2, which corresponds to the p100/p52 subunits of NF-κB, responsible for the activation of the non-canonical NF-κB pathway, and was found to be significantly more abundant in all groups. NFKB1, on the other hand, which corresponds to the NF-κB subunits p105/p50 and is responsible for the activation of the canonical NF-κB pathway, was found to be significantly more abundant in the TNF and IL-1β groups only. Vimentin (VIM), a cytoskeletal protein with a key role during astrogliosis, and cadherin-2 (CDH2), a protein upregulated during scar formation [32], belonged to this cluster and were only found significantly more abundant in the TNF group.

Most of the proteins belonging to cluster 3 were affected by IL-1β, only in part by TNF, and not at all by LPS. Among these, we found that the CEBPB transcription factor (also known as NF-IL-6) was significantly more abundant, as were the astrocyte precursor marker CD44, SOD2, and HDGF. Cluster 3 also included some proteins involved in cytoskeleton remodeling, such as MSN, CKAP4, and DPYSL2.

Cluster 4 mostly consisted of proteins which only changed levels significantly in the TNF and LPS groups. Several of these proteins were found to be involved in type I IFN signaling, such as the ISGF3 heterotrimeric transcription factor (consisting of STAT1, STAT2, and IRF9) and the ISG15 ubiquitin-like protein (a cytokine that is transcriptionally regulated by IFN-α and IFN-β) [33], which is known to activate EIF2AK2 (also known as PKR and found significantly more abundantly in the TNF and LPS groups). Other IFN-inducible proteins were ADAR (also known as ADAR1) [34], DTX3L [35], TRIM25 [36], and the antiviral SAMHD1 protein, which has been extensively studied for its role in HIV infection [37, 38].

Finally, clusters 5 and 6 included proteins that significantly decreased their abundance upon TNF and IL-1β exposure and involved in cytoskeleton remodeling, such as keratins (KRT7, KRT8, KRT18), but also proteins belonging to the extracellular matrix, such as collagens (COL1A1, COL1A2, COL18A1) and laminins (LAMB1, LMNA).

### Cytokine secretion upon TNF, IL-1β, and LPS exposure

**Fig. 3:**
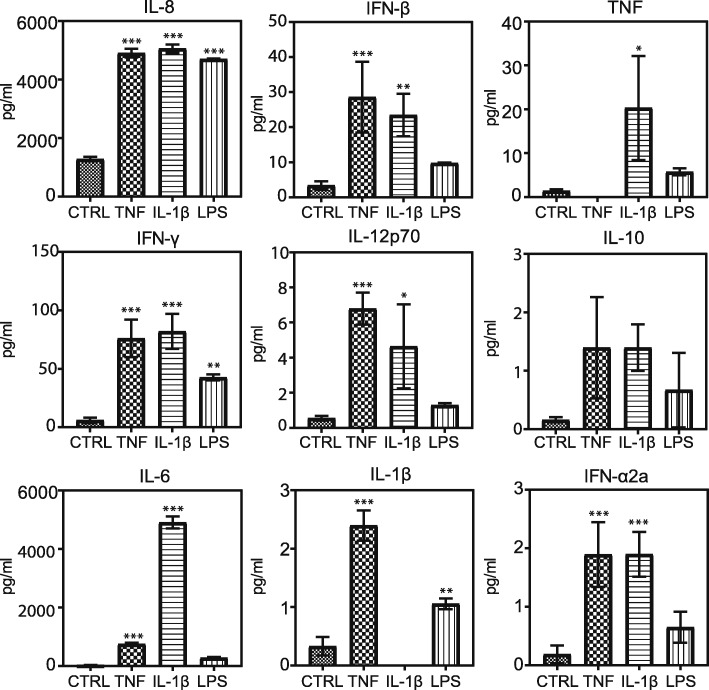
Supernatant cytokine and chemokine concentrations upon stimulating cells with TNF (100 ng/ml), IL-1β (100 ng/ml), and LPS (10 μg/ml) for 24 h. Data are represented as means ± SD of three biological replicates. One-way ANOVA with Dunnet’s post hoc test was used to test for significance in each group compared to the control (*P < 0.05, **P < 0.01, and ***P < 0.005)

The analysis of supernatants revealed that both TNF and IL-1β significantly increased the secretion of IFN-α2a, IFN-β, IFN-γ, IL-12p70, IL-1β, IL-6, IL-8, and TNF (Fig. 3). IL-10 was the only molecule tested that was not found secreted at statistically different levels. TNF and IL-1β induced similar magnitudes of secretion in almost all of the molecules tested, except IL-6, which was far more substantially secreted upon IL-1β exposure than upon TNF exposure.

LPS exposure only significantly increased the secretion of IFN-γ, IL-1β, and IL-8. IL-8 was found at very similar concentrations in the supernatants of all the exposure groups, whereas concentrations of IFN-γ and IL-1β were lower in LPS group’s supernatant.

### LPS-induced activation of the non-canonical NF-κB pathway in immortalized human astrocytes

**Fig. 4:**
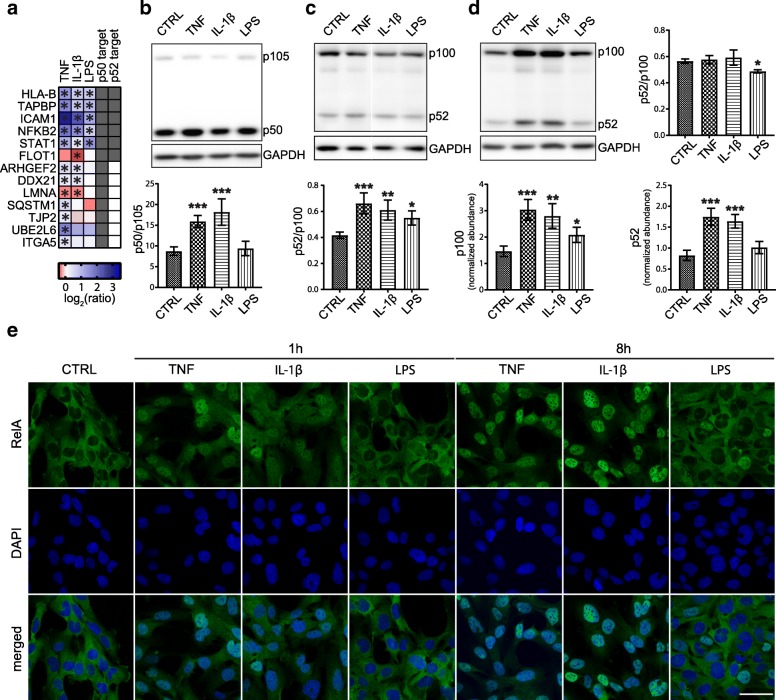
LPS activated NF-κB through the non-canonical pathway. (**a**) Significantly changing proteins were compared with known NFKB1 p50 and NFKB2 p52 targets [39]. Western blotting analysis for NFKB1 p105/p50 (**b**) and NFKB2 p100/p52 (**c**) following a 1-h exposure to TNF, IL-1β, and LPS. (**d**) Western blotting analysis for NFKB2 p100/p52 following a 24-h exposure and plot for p100/p52 ratio and abundances of p100 and p52. Protein abundances were normalized using the intensity of GAPDH. Blots are representative of three biological replicates, and data are represented as means ± SD. One-way ANOVA with Dunnet’s post hoc test was used to test for significance in each group compared to the control (*P < 0.05, **P < 0.01, and ***P < 0.005). (**e**) Confocal images of cells immuno-labeled for RelA following a 1-h or 8-h exposure to TNF, IL-1β, and LPS. Nuclei were counterstained with DAPI. The images are representative of two biological replicates. Scale bar, 50 μm

DIA MS data indicated that LPS exposure increased the abundance of NFKB2 and several other proteins, such as STAT1, ICAM1, FLOT1, TAPBP, and HLA-B, which are targets of NFKB2 p52 [39] (Fig. 4a), suggesting that LPS-induced activation of NF-κB may be mediated predominantly via the non-canonical pathway.

This hypothesis was tested by analyzing NFKB1 p105 and NFKB2 p100 processing and the nuclear translocation of the RelA subunit upon exposure to TNF, IL-1β, and LPS. As expected, following 1-h exposure, TNF and IL-1β induced significant processing of p105, while LPS did not (Fig. 4b). Instead, 1-h exposure to TNF, IL-1β, and LPS induced significant p100 processing, as shown by the increase in the p52/p100 ratio (Fig. 4c). After a 24-h exposure, the p52/p100 ratio was not found to have significantly changed from the control level in TNF and IL-1β groups, whereas it was slightly, but significantly, lower in the LPS group (Fig. 4d). In addition, 24-h exposure increased the abundance of p100 in all three groups, whereas p52 abundance increased in the TNF and IL-1β groups only. Confocal images revealed that LPS was unable to induce nuclear translocation of RelA during a 1-h exposure, and only a few nuclei had been positively stained with RelA after an 8-h exposure (Fig. 4e).

### LPS-induced activation of primary human astrocytes in an in vitro model of BBB

**Fig. 5:**
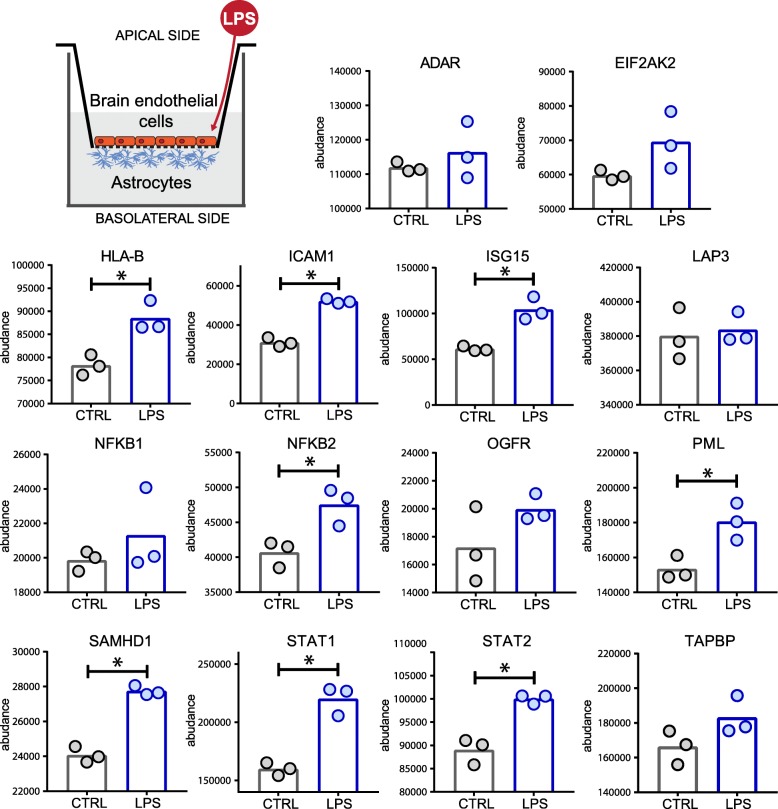
LPS-induced astrocyte activation in an in vitro model of BBB. Primary human astrocytes were grown in co-culture with primary HBMECs. The BBB model was exposed to LPS (100 ng/ml) for 24 h. The protein content of astrocytes was analyzed by DIA MS. Asterisks indicate significance levels (*P* < 0.05)

We then aimed at verifying the modulation of the abundance of several inflammatory-related proteins in primary human astrocytes in a more complex in vitro setting. Therefore, we developed an in vitro BBB model by co-culturing a monolayer of primary human brain microvascular endothelial cells (HBMECs) together with primary human astrocytes (HAs) using a Transwell system (Fig. 5). We subsequently exposed the model to LPS for 24 h, collected the astrocytes from the basolateral side, and quantified their protein content by DIA MS. Samples were analyzed using a spectral library (7642 proteins, 54,193 peptides) generated from 12 injections from fractionated cell lysates derived from the primary human astrocytes.

The results (Fig. 5) showed that LPS-induced inflammatory response involved several type I IFN signaling-related proteins such as STAT1, STAT2, ISG15, and SAMHD1 but not others such as ADAR or EIF2AK2. NFKB2 was found significantly more abundant while NFKB1 was not. Finally, other proteins which have important roles in inflammation such as ICAM1, HLA-B, and PML were also significantly more abundant upon LPS exposure.

## Discussion

In the present study, we used high-throughput MS-based proteomics to extensively characterize the proteomic inflammatory phenotype of human astrocytes using immortalized astrocytes exposed to TNF, IL-1β, and LPS and primary astrocytes grown in an in vitro BBB model exposed to LPS.

In immortalized astrocytes, TNF, IL-1β, and LPS induced proteome perturbations that converged toward the activation of the non-canonical NF-κB pathway and MHC class I antigen presentation. Activation of NF-κB via the non-canonical pathway has been associated with exposure to LPS [40] and bacteria [41], and it is well known to be induced by TNF and IL-1β in other cell types [42]. However, in most of these studies, the non-canonical pathway is activated in conjunction with the canonical NF-κB pathway, and several lines of evidence propose that NFKB2 p100/p52 is a negative regulator of the canonical NF-κB pathway [43–45]. The present results suggested that LPS predominantly activated NF-κB via the non-canonical pathway, and we hypothesized that this activation could be responsible for regulating the abundance of proteins such as STAT1, ICAM1, and TAPBP, which are NFKB2 p52 targets [39]. Activation of the non-canonical NF-κB pathway usually entails slower kinetics than the canonical pathway since it requires TRAF3 degradation and NIK stabilization and accumulation [46], even though rapid processing of p100 has been reported in some studies [47, 48]. The present study revealed a relatively rapid processing of p100 into p52 after only 1 h of exposure to TNF, IL-1β, or LPS. Our results also suggested that activation of the non-canonical NF-κB induced a positive feedback as the abundance of p100 was significantly increased upon 24-h exposure.

In immortalized astrocytes, TNF and IL-1β induced an overall more potent inflammatory response that involved differential regulation of biological pathways linked to cell adhesion and cytoskeletal remodeling.

In astrocytes, the modulation of cytoskeletal proteins is of particular importance during inflammation since they are responsible for processes leading to scar formation. We found the abundance of several intermediate filament (IF) proteins and cytoskeletal-related proteins, both increased (VIM, MSN) and decreased (PALLD, EZR, PLEC, KRT8, KRT18). GFAP, one of the most important IF proteins found in mature astrocytes and associated with astrogliosis, was not quantified by mass spectrometry in immortalized cells, probably because of its low abundance in human fetal astrocytes, as shown elsewhere [49].

We found that cell adhesion molecules (CAMs), such as ICAM1, BCAM, CD44, ITGA1, and ITGA5, were significantly changing their abundance under inflammatory conditions. CAMs in astrocytes are important for the molecular assembly of newly formed synapses and for facilitating interaction with lymphocytes, ultimately leading to the recruitment of immune cells in the CNS during inflammation [50]. CD44, a receptor for hyaluronic acid (HA), a key component of the brain’s extracellular matrix, is thought to play important roles in axon guidance during neuronal development, and its upregulation has been associated to astrogliosis [10] and neurodegenerative diseases [51, 52].

We also found that several type I interferon-inducible proteins, such as EIF2AK2, ADAR, TRIM25, and ISG15, were significantly more abundant upon TNF and LPS exposure, but not upon IL-1β exposure. LPS is commonly known to induce IFN-β and IFN-inducible genes through the TRIF-dependent pathway [53], although our results did not suggest a significant increase in IFN-β secretion upon LPS exposure. TNF and LPS significantly increased the abundance of STAT1, STAT2, and IRF9. Tyrosine-phosphorylated STAT1 and STAT2, together with IRF9, are commonly known to form the tripartite transcription factor IFN-stimulated gene factor 3 (ISGF3) complex—a transcription factor that, following IFN-β exposure, binds interferon-stimulated genes (ISGs) [54]. However, it has been shown that un-phosphorylated ISGF3 (U-ISGF3) formed by IFN-β-induced high levels of IRF9, STAT1, and STAT2 (without tyrosine phosphorylation) is also able to induce the expression of some antiviral genes such as IFI27, BST2, OAS1, OAS2, OAS3, and STAT1 but unable to induce expression of IFN-α and IFN-β [55, 56]. These findings may explain our results, and they suggest that TNF- and LPS-induced expression of U-ISGF3 upregulated few type I IFN-inducible proteins. Nevertheless, further investigations would be needed to confirm this hypothesis.

TNF and IL-1β significantly increased the secretion of all tested cytokines and chemokines apart from IL-10, consistently with previous studies reporting the TNF/IL-1β-induced secretome in human astrocytes [57]. Instead, LPS only induced significant upregulated secretion of IFN-γ, IL-1β, and IL-8. IL-8 was the sole molecule that was secreted at similar concentrations upon exposure to all three stimuli, while LPS-induced secretion of IFN-γ and IL-1β was less potent compared to TNF and IL-1β. This difference between TNF, IL-1β, and LPS may be linked to the activation of canonical NF-κB by TNF and IL-1β, as all the molecules tested are known NFKB1 p50 targets [58]. However, little is known about how non-canonical NF-κB participates in regulating the transcription of these molecules, although NFKB2 has been shown to negatively regulate type I IFN induction in mice [59]. Overall, TNF and IL-1β modulated the secretion of all the tested cytokines and chemokines similarly, except for IL-6, which was more potently secreted upon IL-1β exposure. IL-6 is a pro- and anti-inflammatory cytokine associated with TNF and IL-1β exposure, it is involved in glial scar formation, and its secretion by astrocytes is correlated with reduced BBB function [60]. IL-6 transcription is known to be regulated by both NFKB1 p50 and CEBPB [61]. CEBPB, an important transcription factor that regulates the expression of several IL-1β-induced genes [62], was found to be significantly more abundant upon exposure to IL-1β, but not to TNF and may therefore be responsible for part of the observed differences between TNF and IL-β.

Our results are partially in contrast with some studies on human astrocytes that report no significant increase in secretion of cytokines and chemokines following exposure to LPS [21, 63] but consistent with other studies reporting modest LPS-induced secretion of cytokines in this cell type [64]. These differences may arise from the differences in LPS concentrations that were used among studies, the sensibility of the assays, and the differences in the type of astrocytes used (fetal vs mature, primary cells vs cell lines as well as the region of the brain from where they were isolated). Astrocytes are known for their protective and supportive role inside the brain thanks to the secretion of a wide range of active molecules able to modulate synapse development, neuronal activity and plasticity, and BBB physiology. Significant perturbations in the secretion of these molecules can therefore have an active impact on the physiology of the CNS. We found proteins involved in CNS synaptogenesis (THBS1) [65], angiogenesis inhibition (COL18A1) [66], and BBB formation (AHNAK) [67] significantly decreased in abundance upon TNF and IL-1β exposure.

We decided to use a primary human cell-based in vitro BBB model to study the inflammatory response of astrocytes in a more close to in vivo situation. We showed that in this context, primary astrocytes responded to LPS with similar patterns of activation compared to what was observed in immortalized astrocyte monoculture by increasing the abundance of proteins associated with the type I IFN (STAT1, STAT2, ISG15) and the non-canonical NF-κB (NFKB2) signaling. Obviously, in this context, the inflammatory response observed in astrocytes in co-culture is at least partially attributable to paracrine signaling between astrocytes and HBMECs.

Most of the biological mechanisms that mediate inflammatory responses induced by cytokines and endotoxins are strictly regulated by positive and negative feedback that can sustain or mitigate biological effects. Also, NF-κB activation kinetics can change depending on the activation stimulus, generating unique biological responses [68]. It is important to note, therefore, that the present study reports a snapshot of the proteome under the inflammatory conditions induced by 24 h of exposure to TNF, IL-1β, or LPS. Furthermore, most of the experiments were performed using immortalized human fetal astrocytes. Because adult human and fetal human astrocytes have been shown to possess different traits and respond differently to stimuli [69], we cannot exclude that some of the observations reported are not representative of adult human astrocytes.

## Conclusions

We used DIA MS to systematically identify the proteomic inflammatory phenotype of human astrocytes using both immortalized fetal astrocytes exposed to TNF, IL-1β, and LPS, as well as primary human astrocytes co-cultured with HBMECs and exposed to LPS.

We reported distinct clusters of proteins whose abundance significantly changed upon exposing immortalized astrocytes to the different inflammatory stimuli and described the associated downstream biological pathways. TNF and IL-1β both activated the canonical and non-canonical NF-κB and induced a severe inflammatory response that ultimately led to differential expression of several cytoskeletal and adhesion proteins. Instead, astrocytes responded to LPS by activating the non-canonical NF-κB pathway, and by increasing the abundance of several interferon-stimulated gene products. Finally, similar responses were observed in primary human astrocytes cultured in an in vitro model of the BBB exposed to LPS.

Since astrocytes are crucial regulators of innate and adaptive immune responses in the CNS and their dysregulation and activation is associated with most neurodegenerative disorders, unveiling their proteomic profile under inflammation is important for better understanding their pathophysiological state and their role in the central nervous system in health and disease.

## Additional files

Additional file 1: Chromatographic gradient profile used for LC-MS analysis. (DOCX 12 kb)

Additional file 2: Isolation windows used for DIA MS analysis. (DOCX 15 kb)

Additional file 3: Effects of 24-h exposure to TNF (100 ng/ml), IL-1β (100 ng/ml), and LPS (10 μg/ml) on cell death and viability assessed using LDH and MTS assays, respectively. Data are represented as means ± SD of three biological replicates. (DOCX 143 kb)

Additional file 4: Pearson correlation coefficients of peptide intensities in all 15 samples compared with each other. (DOCX 276 kb)

Additional file 5: List of proteins that were found significantly changing (FDR < 0.05, |FC| > 1.2) under at least one condition (TNF, IL-1β, and LPS). (XLSX 33 kb)

Additional file 6: Correlation of ratios of significantly changing proteins shared between TNF and IL-1β groups. (DOCX 46 kb)

Additional file 7: Biological pathways associated with each cluster of proteins. (XLSX 13 kb)

## Abbreviations

AD: Alzheimer’s disease
BBB: Blood-brain barrier
BSA: Bovine serum albumin
CNS: Central nervous system
DDA: Data-dependent acquisition
DIA: Data-independent acquisition
FDR: False discovery rate
GFAP: Glial fibrillary acidic protein
HBMECs: Human brain microvascular endothelial cells
IPG: Immobilized pH gradient
ISGF3: IFN-stimulated gene factor 3
ISGs: Interferon-stimulated genes
LC-ESI-MS/MS: Liquid chromatography-electrospray ionization-MS/MS
LDH: Lactate dehydrogenase
LPS: Lipopolysaccharide
MS: Mass spectrometry
OGE: Off-Gel electrophoresis
PCA: Principal component analysis
PD: Parkinson’s disease
PFA: Paraformaldehyde
PSMs: Peptide spectrum matches
U-ISGF3: Un-phosphorylated ISGF3
