# Supplementary material for: Profiling the proteomic inflammatory state of human astrocytes using DIA mass spectrometry
Source: J Neuroinflammation. 2018 Nov 30;15:331. doi: 10.1186/s12974-018-1371-6 (PMC6267034; doi:10.1186/s12974-018-1371-6)
Supplement: Supplementary file 1 — Chromatographic gradient profile used for LC-MS analysis. (DOCX 12 kb) [file 12974_2018_1371_MOESM1_ESM.docx]

| %B | Time (min) | Duration (min) |
| --- | --- | --- |
| 8 | 0 | 0 |
| 28 | 105 | 105 |
| 42 | 125 | 20 |
| 95 | 135 | 10 |
| 95 | 160 | 25 |
