# Supplementary material for: Profiling the proteomic inflammatory state of human astrocytes using DIA mass spectrometry
Source: J Neuroinflammation. 2018 Nov 30;15:331. doi: 10.1186/s12974-018-1371-6 (PMC6267034; doi:10.1186/s12974-018-1371-6)
Supplement: Supplementary file 2 — Isolation windows used for DIA MS analysis. (DOCX 15 kb) [file 12974_2018_1371_MOESM2_ESM.docx]

| Method A |  |  |  | Method B |  |  |
| --- | --- | --- | --- | --- | --- | --- |
| Window # | start (m/z) | end (m/z) |  | Window # | start (m/z) | end (m/z) |
| 1 | 399.5 | 419.8 |  | 1 | 399.5 | 423.2 |
| 2 | 418.8 | 431.7 |  | 2 | 422.2 | 438.2 |
| 3 | 430.7 | 444.4 |  | 3 | 437.2 | 452.7 |
| 4 | 443.4 | 455.2 |  | 4 | 451.7 | 466.7 |
| 5 | 454.2 | 467.1 |  | 5 | 465.7 | 480.8 |
| 6 | 466.1 | 478.4 |  | 6 | 479.8 | 494.4 |
| 7 | 477.4 | 489.7 |  | 7 | 493.4 | 507.3 |
| 8 | 488.7 | 501.2 |  | 8 | 506.3 | 519.8 |
| 9 | 500.2 | 510.8 |  | 9 | 518.8 | 532.4 |
| 10 | 509.8 | 520.6 |  | 10 | 531.4 | 545.4 |
| 11 | 519.6 | 530.3 |  | 11 | 544.4 | 558.3 |
| 12 | 529.3 | 540.5 |  | 12 | 557.3 | 571.4 |
| 13 | 539.5 | 550.7 |  | 13 | 570.4 | 585.5 |
| 14 | 549.7 | 560.8 |  | 14 | 584.5 | 599 |
| 15 | 559.8 | 571.3 |  | 15 | 598 | 613.1 |
| 16 | 570.3 | 581.3 |  | 16 | 612.1 | 627.5 |
| 17 | 580.3 | 591.8 |  | 17 | 626.5 | 642.3 |
| 18 | 590.8 | 601.8 |  | 18 | 641.3 | 658.4 |
| 19 | 600.8 | 611.8 |  | 19 | 657.4 | 675.4 |
| 20 | 610.8 | 622.8 |  | 20 | 674.4 | 694.2 |
| 21 | 621.8 | 633.8 |  | 21 | 693.2 | 714.3 |
| 22 | 632.8 | 645.6 |  | 22 | 713.3 | 736.6 |
| 23 | 644.6 | 657.1 |  | 23 | 735.6 | 761.4 |
| 24 | 656.1 | 668.5 |  | 24 | 760.4 | 787.6 |
| 25 | 667.5 | 680.4 |  | 25 | 786.6 | 817.9 |
| 26 | 679.4 | 693.2 |  | 26 | 816.9 | 850.8 |
| 27 | 692.2 | 707.5 |  | 27 | 849.8 | 892.4 |
| 28 | 706.5 | 722.9 |  | 28 | 891.4 | 952.9 |
| 29 | 721.9 | 739.4 |  | 29 | 951.9 | 1046.6 |
| 30 | 738.4 | 755.9 |  | 30 | 1045.6 | 1250.5 |
| 31 | 754.9 | 774 |  |  |  |  |
| 32 | 773 | 792.9 |  |  |  |  |
| 33 | 791.9 | 815.9 |  |  |  |  |
| 34 | 814.9 | 838.9 |  |  |  |  |
| 35 | 837.9 | 864 |  |  |  |  |
| 36 | 863 | 895.6 |  |  |  |  |
| 37 | 894.6 | 935.3 |  |  |  |  |
| 38 | 934.3 | 994.3 |  |  |  |  |
| 39 | 993.3 | 1076.7 |  |  |  |  |
| 40 | 1075.7 | 1250.5 |  |  |  |  |
