# Supplementary figures and images for: Profiling the proteomic inflammatory state of human astrocytes using DIA mass spectrometry
Source: J Neuroinflammation. 2018 Nov 30;15:331. doi: 10.1186/s12974-018-1371-6 (PMC6267034; doi:10.1186/s12974-018-1371-6)

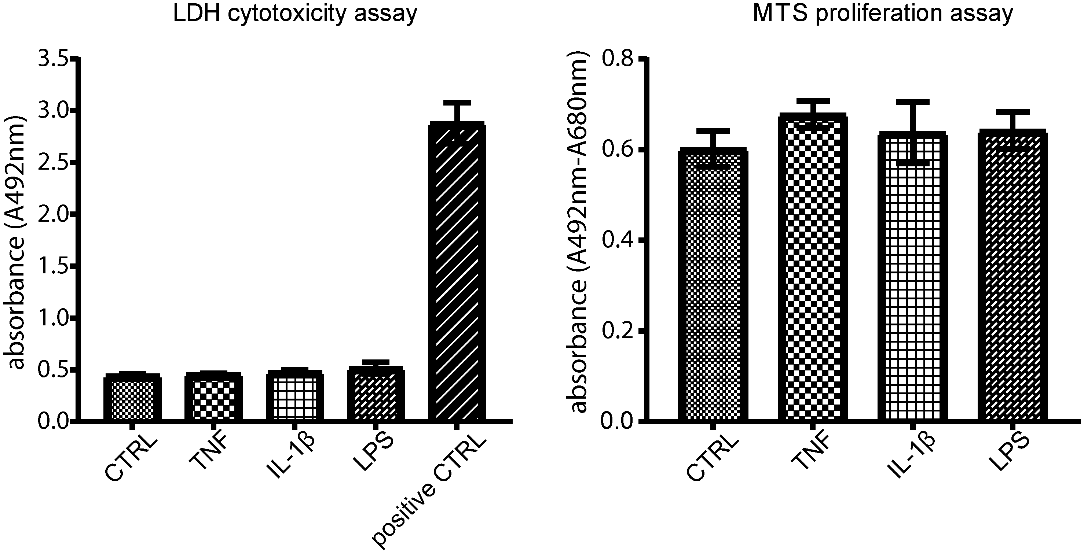

Supplement: Supplementary file 3 — Effects of 24-h exposure to TNF (100 ng/ml), IL-1β (100 ng/ml), and LPS (10 μg/ml) on cell death and viability assessed using LDH and MTS assays, respectively. Data are represented as means ± SD of three biological replicates. (DOCX 143 kb) [file 12974_2018_1371_MOESM3_ESM.docx]

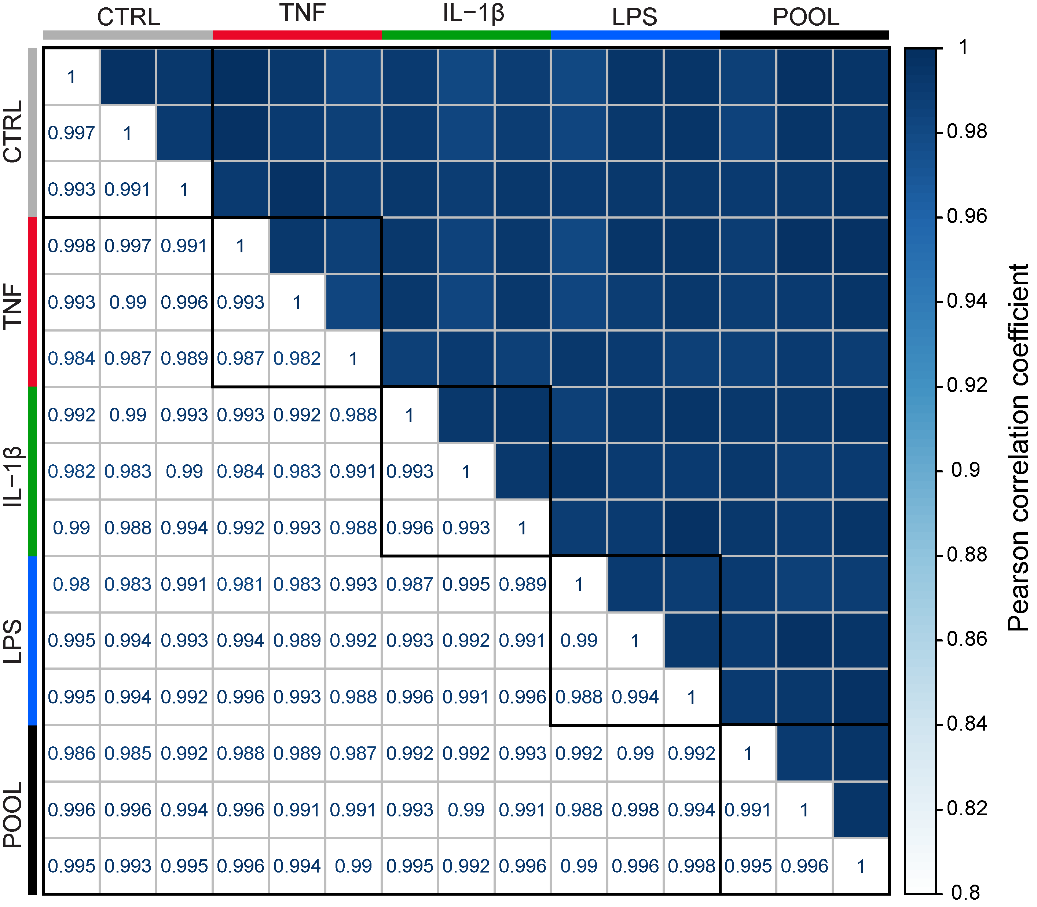

Supplement: Supplementary file 4 — Pearson correlation coefficients of peptide intensities in all 15 samples compared with each other. (DOCX 276 kb) [file 12974_2018_1371_MOESM4_ESM.docx]

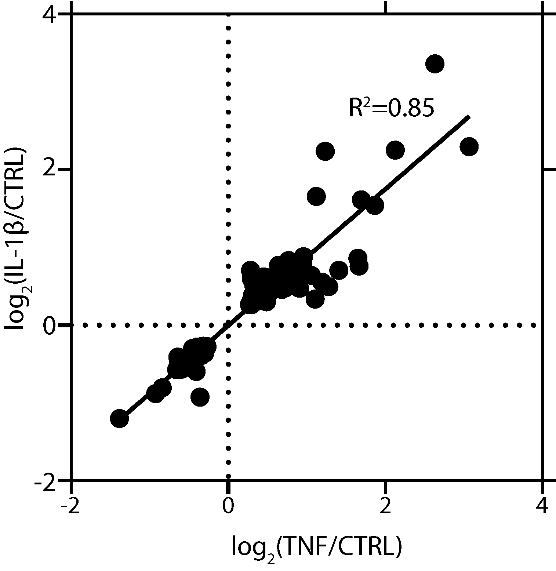

Supplement: Supplementary file 6 — Correlation of ratios of significantly changing proteins shared between TNF and IL-1β groups. (DOCX 46 kb) [file 12974_2018_1371_MOESM6_ESM.docx]
